# Systematic analysis of expression signatures of neuronal subpopulations in the VTA

**DOI:** 10.1186/s13041-019-0530-8

**Published:** 2019-12-11

**Authors:** Hyun Jin Kim, Minhyung Kim, Byeongsoo Kang, Soyeon Yun, Shin Eun Ryeo, Daehee Hwang, Joung-Hun Kim

**Affiliations:** 1 Department of Life Sciences, Pohang University of Science and Technology, Pohang, Republic of Korea; 2 Departments of Surgery and Biomedical Sciences, Cedars-Sinai Medical Center, Los Angeles, CA USA; 3 Department of Biological Sciences, Seoul National University, Seoul, Republic of Korea

**Keywords:** Allen brain atlas, Gene expression profile, VTA, Cell type markers

## Abstract

Gene expression profiling across various brain areas at the single-cell resolution enables the identification of molecular markers of neuronal subpopulations and comprehensive characterization of their functional roles. Despite the scientific importance and experimental versatility, systematic methods to analyze such data have not been established yet. To this end, we developed a statistical approach based on in situ hybridization data in the Allen Brain Atlas and thereby identified specific genes for each type of neuron in the ventral tegmental area (VTA). This approach also allowed us to demarcate subregions within the VTA comprising specific neuronal subpopulations. We further identified WW domain-containing oxidoreductase as a molecular marker of a population of VTA neurons that co-express tyrosine hydroxylase and vesicular glutamate transporter 2, and confirmed their region-specific distribution by immunohistochemistry. The results demonstrate the utility of our analytical approach for uncovering expression signatures representing specific cell types and neuronal subpopulations enriched in a given brain area.

## Introduction

The brain is an extremely complicated organ containing myriad regions for the distinct processing and integration of neural information. These regions are composed of diverse subregions, only some of which have been characterized thus far. To understand the functional roles of individual neural circuits, the primary resident neuron types must first be identified. Conventionally, neuron types have been classified in accordance with their morphology, connectivity, and electrophysiological features [1–3]. There is a limited set of established markers for neuron types, and the expression patterns of many genes remain uncharacterized [4]. Currently, in situ hybridization (ISH) data are available in the Allen Brain Atlas (ABA), providing brain-wide gene expression profiles in adult mice, particularly at the single-cell resolution [5, 6]. The ISH data provide opportunities to search and pinpoint genes that were selectively expressed in neuronal subpopulations [7, 8]. The select genes can then serve as molecular signatures that represent these neurons.

ISH data in the ABA have been used to identify neuronal subpopulations whose functions were investigated with genetic animal models. For instance, *Elfn1* is expressed by subpopulations of interneurons within the oriens-lacunosum moleculare area of the hippocampus and confers target-specific synaptic properties [9]. Hence, the identification of the neuronal subpopulation by a marker gene led to the functional characterization of the subregion in which they mainly reside. However, the ISH data are not in an easily accessible format, which would deter systematic searches for genes expressed specifically in subpopulations.

The ventral tegmental area (VTA) is a midbrain dopamine-producing center that is causally involved in emotional states such as motivation and reward [10, 11]. The VTA largely comprises dopaminergic, glutamatergic, and GABAergic neurons that express the key enzymes for the synthesis and release of their respective neurotransmitters [12, 13]. However, it is not clear whether cellular identity can be systematically analyzed by profiling gene expression in each subregion of the VTA or which genes are selectively expressed by each cell type. To address these questions, we developed and applied analytical approaches for identifying molecular markers of the neuronal subpopulations enriched in VTA subregions. This newly developed experimental algorithm provided a set of unanticipated genes as molecular markers of VTA cell types.

## Materials and methods

### Identification of alternative marker genes

To identify potential marker genes for glutamatergic, dopaminergic, and GABAergic neurons in the VTA, for 1143 genes with the data available, Spearman’s correlations of their expression intensities in the 42 voxels of the VTA were calculated with the expression intensities of the following three known maker genes: tyrosine hydroxylase (TH; the enzyme required for dopamine synthesis), vesicular glutamate transporter 2 (VGLUT2; encoded by *Slc17a6*), and glutamate decarboxylase 67 (GAD67; encoded by *Gad1*). *P* values of the correlations between the genes and those known marker genes for the null hypothesis (i.e., gene is not correlated with the markers) were estimated according to a *t* test [14] previously described for the correlation coefficient. The correlations with *P* < 0.05 were considered to be statistically significant, and thereby marker candidates were selected as the genes with significant positive correlations uniquely with a known marker gene. Those genes having significant positive correlations with each used marker gene could show significant (*P* < 0.05) negative correlations with the other marker genes. On the basis of the correlation patterns (positive, negative, or no significant correlation) with the known marker genes, the selected candidate genes were grouped into 11 clusters. The final marker candidates were genes that correlated positively with the neuron type of interest, but correlated negatively with the other two neuronal types.

### Identification of marker genes for neurons co-releasing dopamine and glutamate

A virtual expression profile of a marker gene for neurons co-releasing dopamine and glutamate was constructed by taking the minimum expression levels of *Th* and *Slc17a6* across the grid voxels of the VTA, assuming that these values would be the maximum expression levels achieved by neurons expressing both *Th* and *Slc17a6*. To identify marker candidates for the co-releasing neurons, Spearman’s correlation values were calculated between the expression profiles of each candidate gene in the grid voxels of the VTA and the virtual expression profile. The *P* value of the correlation was computed according to the *t* test mentioned above. The marker candidate genes for neurons co-releasing dopamine and glutamate displayed positive correlation with a *P* value of < 0.05.

### Animals and tissue preparation

Male C57BL/6 J mice were housed under a 12-h light/dark cycle with ad libitum access to food and water. All procedures for animal experiments were approved by the ethical review committee of POSTECH (Pohang University of Science & Technology), Korea, and performed in accordance with the relevant guidelines. Mice were anesthetized by intraperitoneal injection of Avertin (250 mg/kg body weight, T48402; Sigma) and transcardially perfused with PBS followed by 4% formaldehyde. The brains were isolated, postfixed overnight at 4 °C in a 4% formaldehyde solution, and embedded in 5% agarose gel for sectioning (50-μm-thick coronal sections) with a vibratome (VT1000S; Leica, Germany). Tissue sections containing the VTA region according to the mouse brain atlas [15] were collected.

### Immunohistochemistry

For immunohistochemistry (IHC), prepared tissues were blocked with 4% normal donkey serum and 0.4% Triton X-100 in PBS at 4 °C for 1 h and then incubated with the following primary antibodies at 4 °C overnight: rabbit anti-P2RY14 (1:500, 20,190–1-AP; Proteintech), rabbit anti-CHRNA6 (1:500, GTX51236; GeneTex), rabbit or sheep anti-TH (1:1000, AB152 or AB1542; Millipore), mouse anti-GAD67 (1:500, MAB5406; Millipore), goat anti-VGLUT2 (1:500, ab79157; Abcam), and rabbit anti-WWOX (1:500, sc-366,157; Santa Cruz Biotechnology). Donkey anti-goat DyLight 488-conjugated IgG or donkey anti-sheep DyLight 550-conjugated IgG (1:500; Bethyl Laboratories) and donkey anti-rabbit Alexa 647-conjugated IgG (1:500; Abcam) were used as secondary antibodies. All tissues were mounted on glass slides using UltraCruz mounting medium containing DAPI (Santa Cruz Biotechnology).

### Cellular imaging and quantification

**Fig. 1:**
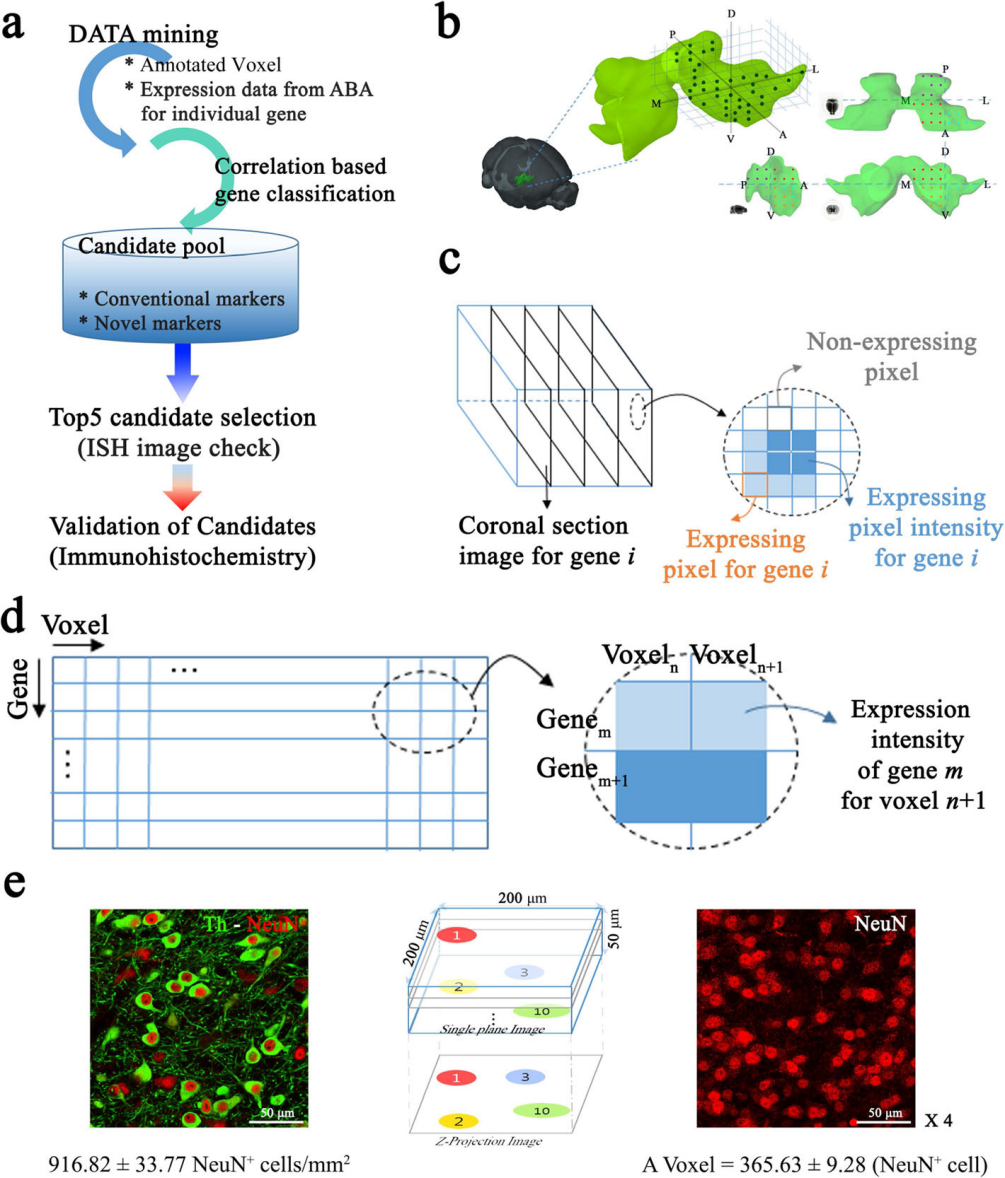
Gene expression profiles across voxels in the VTA. (**a**) Overall scheme of the analytical approach involving (i) data acquisition from the ABA, (ii) data analysis (correlation-based gene classification and selection of marker candidates), and (iii) validation of the candidates. (**b**) Spatially annotated voxels encompassing the VTA. The VTA volume is split into the subregions from the center along the AP, ML, and DV axes, and the 42 voxels were assigned into the subregions after mapping the voxels into the VTA volume. Cross-section views (AP-LM, AP-DV, and LM-DV) show how voxels are divided by the AP, ML, and DV axes. Different colors are used to denote the voxels at the four quadrants in the cross-sections. (**c**) Gene expression intensity is estimated for each voxel. For gene *i*, expression intensity was estimated as the sum of expressing pixel intensities divided by the sum of expressing pixels from four ISH images covering a voxel. (**d**) Gene expression matrix for *m* genes and *n* voxels (*m* = 1143 and *n* = 42). Element (*i*, *j*) in the matrix indicates expression intensity for gene *i* and voxel *j*. **e**. Estimation of the cell count in a unit area (left) and a voxel (right). Neuronal cells were labeled with selective neuronal cell marker, NeuN (red) and imaged at 12 distinct sampling points of the VTA region. For cell counting in each voxel, 10 z-stacked images covering a cube of 50 × 200 × 200 μm^3^ (left) were combined to generate a 2-D projected image from which cells were counted, and the cell count was then multiplied by 4. The average numbers of neuronal cells were calculated and used to determine proportional cell populations (3 mice and 10 brain slices, M location: 10 images, L location: 10 images)

**Fig. 2:**
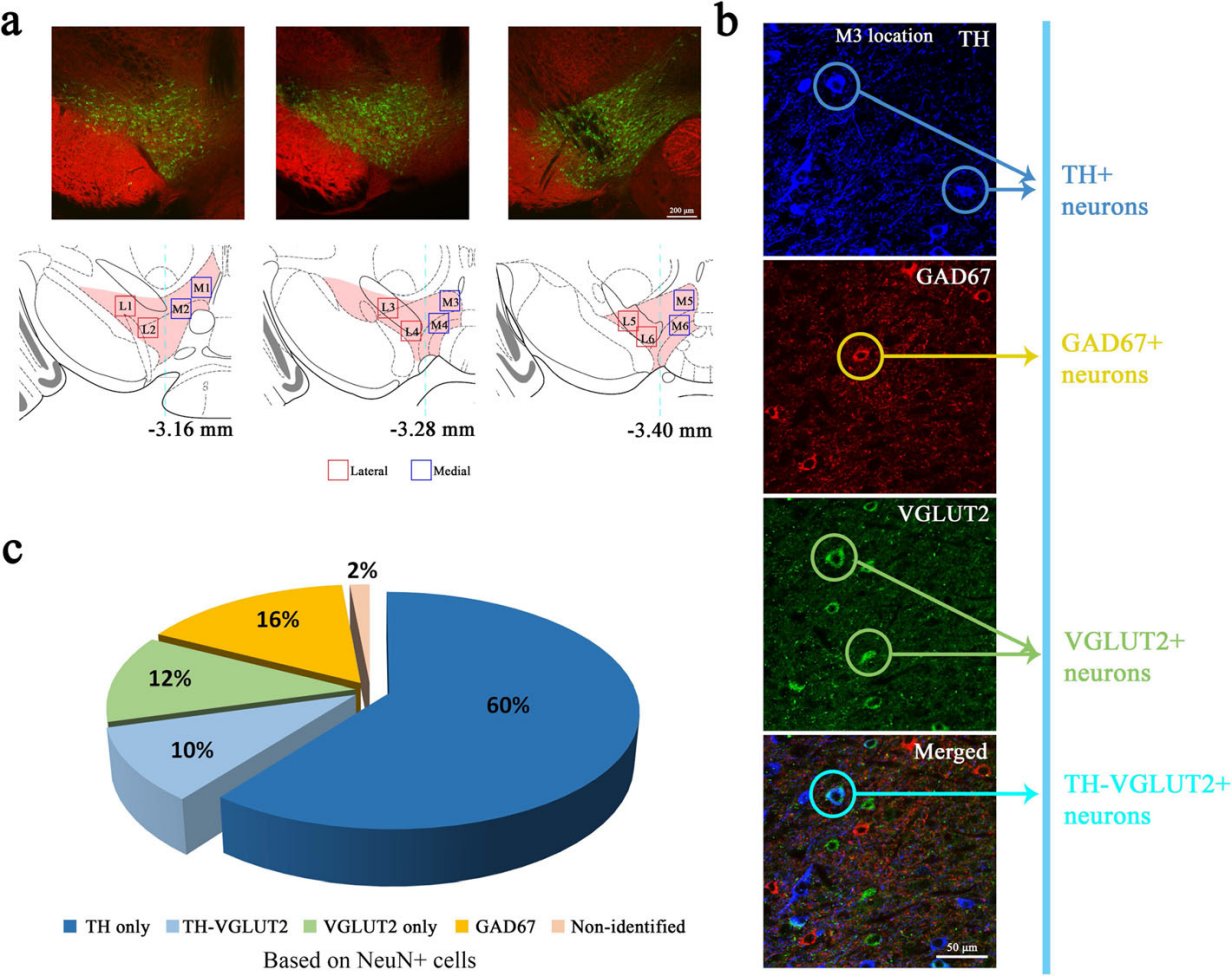
Neuron subpopulations estimated from IHC analysis of the VTA. (**a**) VTA region enriched with dopaminergic neurons (green); red, GAD67 signal. The corresponding atlas maps along the anterior-posterior axis are shown at the bottom (red shading, VTA). Colored squares indicate sampling sites (light blue dotted line indicates medial-lateral separation, 0.5 μm apart from central of the atlas). L1–6 and M1–6 were used to indicate the locations from which the IHC images are obtained. (**b**) Triple immunolabeling for TH^+^ (blue), GAD67^+^ (red), and VGLUT2^+^ (green) neurons in the VTA (4 mice and 13 brain slices, M location: 11 images, L location: 9 images). The representative images were obtained from M3 location in **a**. (**c** Proportions of neuron types in the VTA based on the average number of NeuN^+^ cells (see Fig. 1e; 916.82 ± 33.77 cells/mm^2^)

Sections were imaged with a laser scanning confocal microscope (LSM 510; Zeiss, Germany) with 40× lens objective (C-Apochromat 40 × /1.2 W Korr; scanning area, 230 × 230 μm^2^; image resolution, 1024 × 1024 pixels). Quantitative analysis of immunoreactive signals was performed using MetaMorph 7.7 software (Molecular Devices, Sunnyvale, CA) and Image J (NIH, Bethesda, MD). To estimate the neural cell count within each voxel (200 × 200 × 200 μm^3^), we first counted the cells within a cube of 50 × 200 × 200 μm^3^ as follows (Fig. 1e): 1) we generated 10 z-stacked images (200 × 200 μm^2^) to cover 50 μm in height, 2) we combined these images to generate a 2-D projected image, and 3) then counted cells in the 2-D projected image. We next estimated the cell number in a voxel by multiplying 4 into the cell number counted from 50 × 200 × 200 μm^3^. This procedure was performed for 20 projected images from the 12 distinct sampling locations in the VTA (Fig. 2b), and the average cell count was obtained. In the experiments using different brain slices, we tried to capture all IHC images from the VTA locations indicated in Fig. 2a, which effectively covers the VTA [15]. To further clarify the location information, we assigned IDs to the sampling locations, M1–6 and L1–6, in Fig. 2a and used these IDs to indicate the locations from which the representative images were obtained. Mander’s overlap coefficient was calculated by the Coloc2 plugin function of Image J.

### Experimental design and statistical analysis

For quantification of neurons expressing marker proteins in the VTA, we conducted IHC experiments at the sampling locations of M and L indicated in Fig. 2a. In these experiments, we used the following numbers of animals and images: for NeuN^+^ counting, *N* = 3, 10 brain slices, M location: 10 images, L location: 10 images; for TH-GAD67-VGLUT2 triple labeling, *N* = 4, 13 brain slices, M location: 11 images, L location: 9 images; for TH-CHRNA6 double labeling, N = 3, 11 brain slices, M location: 7 images, L location: 7 images; for VGLUT2-P2RY14 double labeling, N = 3, 11 brain slices, M location: 6 images, L location: 6 images; and for TH-VGLUT2-WWOX triple labeling, *N* = 6, 22 brain slices, M location: 22 images, L location: 14 images).

## Results

### Analytical algorithms for gene expression profiles in the VTA

To parse gene expression profiles in the VTA, we first selected a grid of 42 voxels (200 × 200 × 200 μm^3^) encompassing the VTA according to the annotated three-dimensional reference spaces reconstructed based on ISH and magnetic resonance imaging data in the ABA (Fig. 1a and b). For each gene, expression intensity in each voxel was calculated as the sum of the pixel intensity divided by the sum of expressing pixels from four ISH images (intensity/pixel, Fig. 1c), using the three-dimensional expression grid data. Expression intensities for the 1143 genes available from the coronal section dataset in the 42 voxels were obtained, resulting in a 1143 × 42 gene expression intensity matrix (Fig. 1d). For further cellular quantification, we estimated neuronal cell numbers in brain tissue sections by IHC with a selective neuronal cell marker. Empirically, there were 916.82 ± 33.77 and 365.63 ± 9.28 neuronal cells included in a unit area (mm^2^) and in a voxel (200 × 200 × 200 μm^3^), respectively, in the VTA (Fig. 1e).

### IHC analysis of the VTA

Next, we performed IHC analysis of the VTA using antibodies against TH, VGLUT2, and GAD67 to label dopaminergic, glutamatergic, and GABAergic neurons, respectively. The numbers of each neuron type were counted from 20 images taken at sampling regions along the anterior-posterior axis (indicated in Fig. 2a) to encompass the entire VTA region from multiple mice. GAD67^+^ cells were not largely co-localized with other cell types, but TH^+^ and VGLUT2^+^ cells were partially co-localized (Fig. 2b). The proportions of TH^+^, VGLUT2^+^, and GAD67^+^ neurons were estimated to be 70, 22, and 16%, respectively, of the population of NeuN^+^ cells (set at 100%, see Fig. 1e) (Fig. 2c), which is consistent with previous findings [16, 17]. The remaining 2% of neurons had no detectable expression of TH, VGLUT2, or GAD67. Interestingly, 10% of the neurons expressed both TH and VGLUT2 (see TH-VGLUT2^+^ neuron in Fig. 2b), suggesting that the VTA contains a substantial proportion of neurons that co-release dopamine and glutamate.

### Alternative marker genes to *Th*, *Slc17a6*, and *Gad1*

**Fig. 3:**
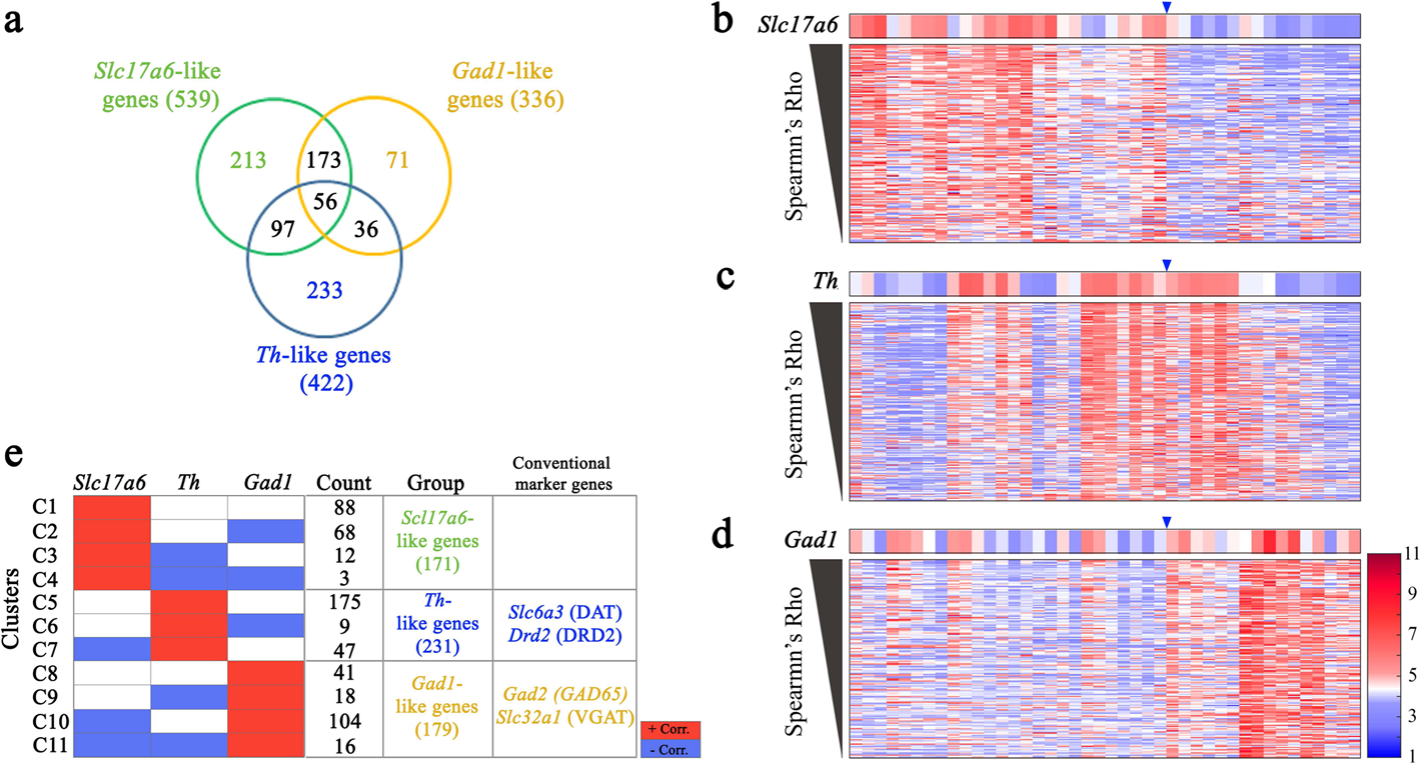
Alternative marker genes to *Slc17a6*, *Th*, and *Gad1*. (**a**) Venn diagram showing relationships among *Slc17a6-*, *Th-*, and *Gad1-*like genes. Numbers of genes belonging to individual clusters are shown. (**b–d**) Heat maps showing similar expression between the selected candidates and *Slc17a6* (**b**), *Th* (**c**), and *Gad1* (**d**). Blue triangles indicate guide points for designation of medial (left, voxels 1–26) and lateral (right, voxels 27–42). Expression intensity for each gene was autoscaled to yield a mean of 0 and a standard deviation of 1 (red, positive; blue, negative). **e** Correlation patterns of *Slc17a6*-, *Th*-, and *Gad1*-like genes. These three groups of genes were categorized into 11 clusters (C1–11) based on their correlations (positive, red; negative, blue) with *Slc17a6*, *Th*, and *Gad1*

To demonstrate the utility of the ISH data in the ABA, we first attempted to identify genes that showed similar expression profiles to the known marker genes, *Th*, *Slc17a6*, and *Gad1*, across the 42-voxel grid in the VTA. To this end, we calculated Spearman’s correlations for the expression intensity of *Th*, *Slc17a6*, or *Gad1* with those of the 1143 genes in the 42 voxels and then estimated the significance (*P* value) of the correlation for each marker gene pair. Using this algorithm, the expression profiles of 539, 422, and 336 genes positively or negatively correlated significantly (*P* < 0.05) with those of *Slc17a6*, *Th*, and *Gad1*, respectively (Fig. 3a). Among these, we selected 171, 231, and 179 genes whose expression intensity patterns were positively correlated uniquely to those of *Slc17a6*, *Th*, and *Gad1*, respectively (Fig. 3b–e). Interestingly, anticorrelations were found between proportions of these genes, which may better distinguish these cell types. For example, among the 231 *Th*-like genes, 47 and 9 showed significant (*P* < 0.05) anticorrelations with *Slc17a6* and *Gad1*, respectively. Similar anticorrelated gene sets were identified from the *Slc17a6*-like genes (68 genes anticorrelated with *Gad1*, 12 genes with *Th*, and three genes with both) and the *Gad1*-like genes (18 genes anticorrelated with *Th*, 104 genes with *Slc17a6*, and 16 genes with both). These genes included previously known marker genes for dopaminergic and GABAergic neurons, namely *Slc6a3* [18, 19] and *Drd2* [18] in *Th*-like genes and *Gad2* [20] and *Slc32a1* [21] in *Gad1*-like genes, respectively (Fig. 3b). These data support the utility of the ISH data in the search for potential marker genes associated with the primary neuron types in the VTA.

### Distributions of distinct neuron types in the VTA

**Fig. 4:**
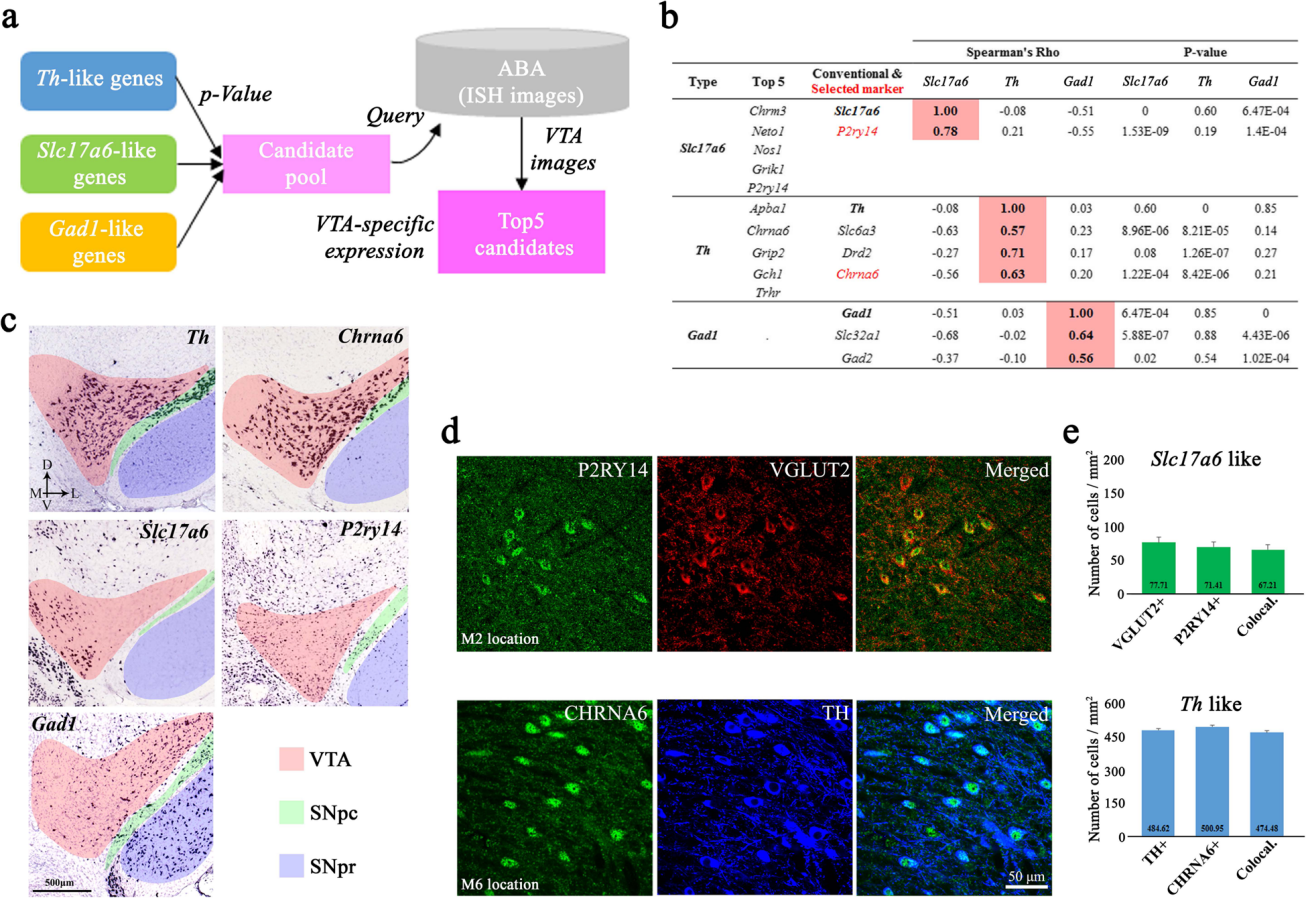
Distribution of neuron subpopulations in the VTA. (**a**) Schematic showing the procedure for selecting the final marker candidates from *Slc17a6-*, *Th-*, and *Gad1-*like genes. See the text for details. (**b**) Top five alternative marker candidates were selected from *Slc17a6-* and *Th-*like genes, and conventional markers were listed with or without a final candidate (red) for each type of neuron. Spearman’s correlations (Rho) are shown, along with their *P* values. (**c**) ISH images showing expression of the indicated genes at the single-cell resolution. Axes: D, dorsal; V, ventral; M, medial; L, lateral. (**d**) Representative IHC images showing expression of the indicated proteins. The images were obtained from M2 and M6 locations in Fig. 2a for visualizing of P2RY14-VGLUT2 labeling and CHRNA6-TH labeling, respectively. (**e**) Numbers of neurons expressing the indicated proteins (3 mice and 11 brain slices per marker candidate; for *Slc17a6* like [M location: 6 images, L location: 6 images], for *Th* like [M location: 7 images, L location: 7 images] used for analysis). Data are shown as mean ± SEM. *Slc17a6*-like: VGLUT2^+^, 77.71 ± 19.03 cells/mm^2^; P2RY14^+^, 71.41 ± 16.63 cells/mm^2^; double-positive (co-local.), 67.21 ± 14.22 cells/mm^2^; *Th-*like: TH^+^, 484.62 ± 26.90 cells/mm^2^; CHRNA6^+^, 500.95 ± 29.60 cells/mm^2^; co-local., 474.48 ± 14.22 cells/mm^2^

The search for alternative marker genes resulted in novel candidates for *Th*^*+*^, *Slc17a6*^*+*^, and *Gad1*^+^ neurons. We determined whether their expression in the VTA correlated with *Th*, *Slc17a6*, and *Gad1* expression using the ISH images in the ABA and selected the top five novel marker candidates for each neuronal type (Fig. 4a and b). From these results, we selected *Chrna6* and *P2ry14* from the *Th*- and *Slc17a6*-like genes, respectively (Fig. 4b and c) for further analysis; none of the top five *Gad1*-like candidates showed expression patterns similar to that for *Gad1* based on the ISH data.

We further examined the anatomical distribution of *Th*, *Slc17a6*, and *Gad1*, as well as the alternative marker candidates, in the VTA via the ISH images. *Th*^*+*^ and *Chrna6*^+^ neurons were distributed throughout the VTA as well as in the substantia nigra pars compacta area (Fig. 4c, top row). *Slc17a6*^*+*^ and *P2ry14*^*+*^ neurons were enriched in the medial part of the VTA, with *P2ry14* also distributed weakly in the substantia nigra pars reticulata (Fig. 4c, middle row). By contrast, *Gad1*^+^ cells were distributed peripherally around the VTA and in the substantia nigra pars reticulata (Fig. 4c, bottom). These data suggest that the anatomical distribution of neurons expressing the marker genes can potentially be used to identify subregions in the structures in which they reside. To assess the validity of *P2ry14* and *Chrna6* as marker genes, we performed IHC to examine the expression of P2RY14 and CHRNA6 in VGLUT2^+^ and TH^+^ cells (Fig. 4d). Quantification of the numbers of single- and double-positive cells confirmed that the expression of these genes can be used as reliable markers of individual cell types (Fig. 4e). Collectively, the data described above support the utility of our analytical approach for identifying marker genes for neuronal subpopulations as well as their distribution in the VTA.

### Marker genes for neurons co-releasing dopamine and glutamate

**Fig. 5:**
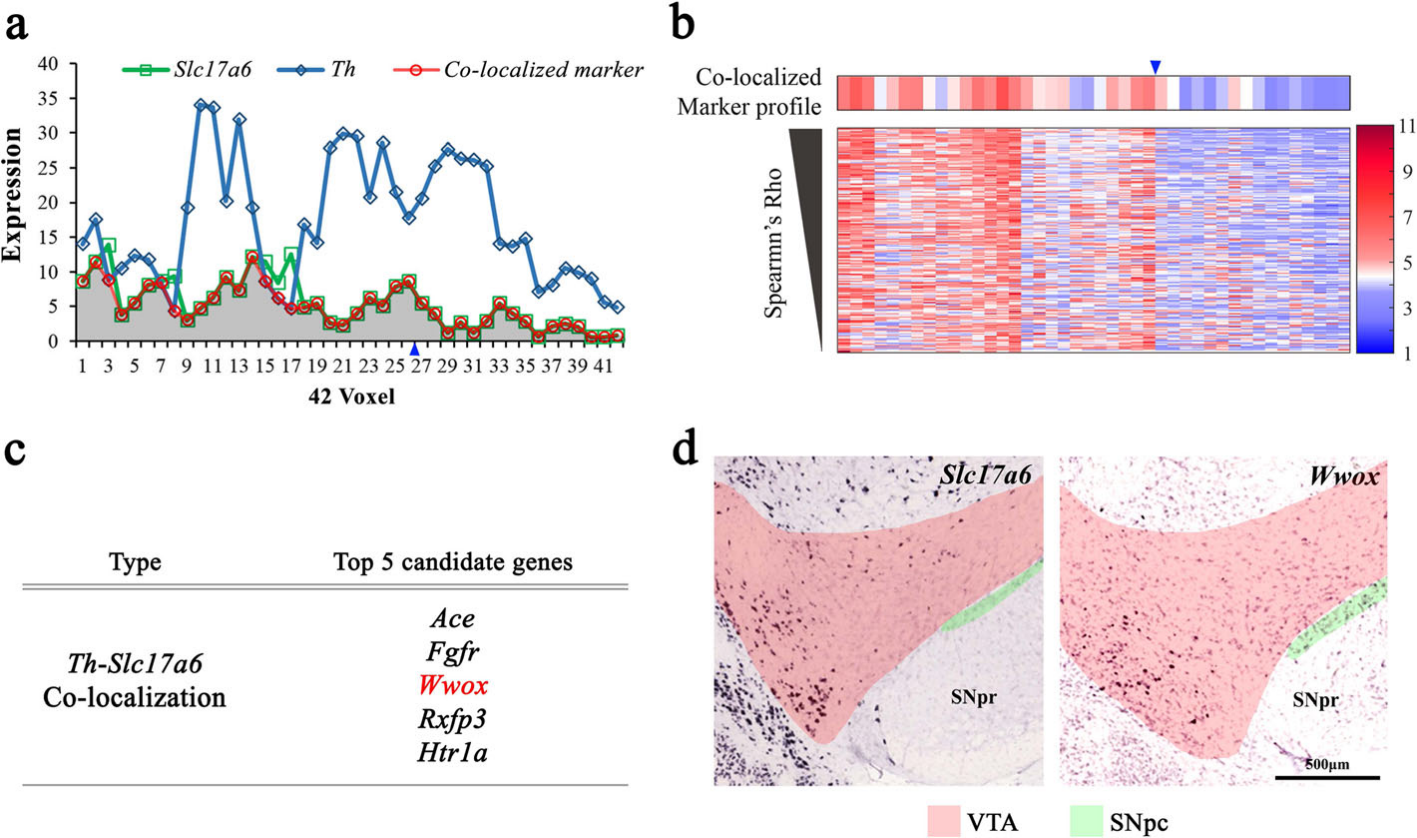
Marker genes for neurons expressing both *Th* and *Slc17a6* in the VTA. (**a**) Virtual expression profile for a marker candidate of neurons expressing both *Th* and *Slc17a6*. The virtual profile was defined as the minimum (red) *Slc17a6* (green) and *Th* (blue) expression intensity. Gray shading indicates the minimum expression level between *Th* and *Slc17a6*. The blue triangle indicates a guide point for designation of medial and lateral (voxels 1–26 and 27–42, respectively). (**b**) Heat map showing that the expression of the selected candidates is similar to that of the virtual expression profile. Expression intensities for each gene were autoscaled to yield a mean of 0 and a standard deviation of 1 (red, positive; blue, negative). The blue triangle indicates a guide point for medial and lateral as for panel **a**. (**c**) List of selected top five marker candidate genes for *Th-Slc17a6* co-localized cells in the VTA (final selected gene is in red). (**d**) ISH images showing expression of *Slc17a6* and *Wwox* in the VTA

**Fig. 6:**
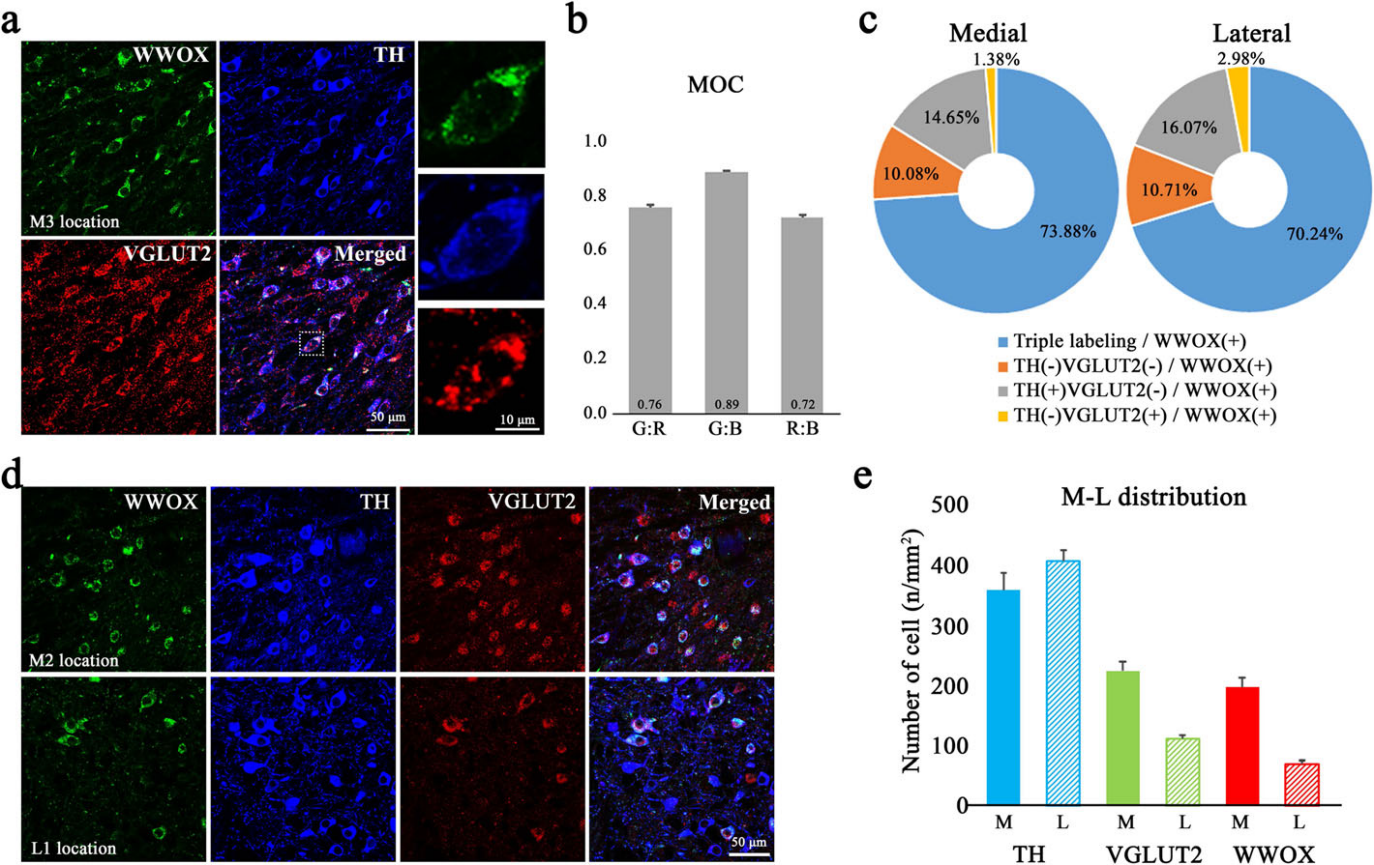
Validation of selected marker gene by IHC analysis. (**a**) Representative IHC images showing expression of the indicated proteins in the medial part of the VTA. The images in the 3rd column show expression of WWOX (green), TH (blue), and VGLUT2 (red) in a subregion indicated by the white dotted box in the merged image. The representative images were obtained from M3 location in Fig. 2a. (**b**) Mander’s overlap coefficient (MOC) analysis was performed on IHC images. Pixel-based overlapped coefficients between green and blue (G:B), green and red (G:R), and red and blue (R:B) channels, *n* = 10 cells. (**c**) Pie charts showing the proportions of WWOX expressing neurons expressing or not expressing TH and/or VGLUT2 in the medial (left) and lateral (right) parts of the VTA (6 mice and 22 brain slices, M location: 22 images, L location: 14 images were used for analysis). (**d**) Representative images showing medial and lateral distributions of neurons expressing WWOX (green), TH (blue) and VGLUT2 (red). These images were obtained from M2 and L1 locations in Fig. 2a. (**e**) Bar graphs showing the numbers of neurons expressing the indicated proteins in the medial (M) and lateral (L) regions of the VTA (image data same as **c**). Data are shown as mean ± SEM. M: TH^+^, 359.17 ± 28.98 cells/mm^2^; VGLUT2^+^, 224.48 ± 14.89 cells/mm^2^; WWOX^+^, 197.31 ± 16.10 cells/mm^2^; L: TH^+^, 407.78 ± 18.02 cells/mm^2^; VGLUT2^+^, 112.07 ± 5.77 cells/mm^2^; WWOX^+^, 70.21 ± 6.08 cells/mm^2^

The IHC analysis confirmed that a subpopulation of neurons in the VTA co-express TH and VGLUT2 (Fig. 2b and c), which can be considered neurons that co-release dopamine and glutamate [13, 16]. Since there are no faithful marker genes for these co-releasing neurons, we sought to examine their gene expression profiles in the VTA. We first computed the minimum expression intensities of *Th* and *Slc17a6* in individual voxels (Fig. 5a, gray shading area), assuming these intensities are the maximum that can originate from neurons co-expressing TH and VGLUT2. Using this idea, we identified 191 genes with expression intensities that correlated significantly (*P* < 0.05) with the minimum intensities of *Th* and *Slc17a6* (Fig. 5b). We then selected the top five candidates (Fig. 5c) and examined ISH images to determine whether they are co-expressed with *Th* and *Slc17a6* in the VTA. We selected the gene encoding WW domain-containing oxidoreductase (*Wwox*), whose expression pattern was most similar to that of *Slc17a6* (Fig. 5d), overlapped with *Th* (Fig. 4c, top left), and was consistent with the minimum expression profiles of *Th* and *Slc17a6* (Fig. 5a). To confirm *Wwox* as a marker of TH- and VGLUT2- co-expressing neurons, we performed an IHC analysis (Fig. 6a) and a pixel-level analysis of fluorescence signals using Mander’s overlap coefficient (Fig. 6a and b). The IHC data showed that > 70% of the neurons that expressed WWOX also expressed both TH and VGLUT2 (Fig. 6c) and were enriched in the medial part of the VTA relative to the lateral part (Fig. 6d and e), which was consistent with the minimum expression profiles of *Th* and *Slc17a6* (Fig. 5a). These data further support the utility of our analytical approach and algorithm in identifying novel marker genes for a subpopulation of neurons and their distribution in the VTA.

## Discussion

In this study, we analyzed gene expression intensities within voxels encompassing the VTA. We estimated from IHC that each voxel contained > 300 neurons and thus may not allow for sufficient spatial resolution in order to pinpoint marker gene expression in individual cells. However, our results demonstrate that such data can provide a list of useful marker candidates, such as *Th* and *Slc17a6* for dopaminergic and glutamatergic neurons, respectively. Our analytical approach suggests that the ISH data can identify marker candidates when the variation in expression intensities across each voxel serves as a representation of the variation in neuron subpopulations in a specific region, such as the VTA.

Our systematic analytical approach involved supervised clustering of the genes based on correlation patterns with the known markers (*Th*, *Slc17a6*, and *Gad1*) to identify alternative markers for neuron subpopulations in the VTA. However, this approach may be not necessary, as we can perform unsupervised clustering of the genes according to the similarity of their expression patterns across the voxels in the grid. Each of the resulting clusters may represent a neuron subpopulation. In this study, unsupervised clustering of the genes within the 42-voxel grids in the VTA using the non-negative matrix factorization method [22] provided four major clusters that included *Th*, *Slc17a6*, *Gad1*, or both *Th* and *Slc17a6*. These results were consistent with those from our supervised clustering approach.

Although dopamine- and glutamate-co-releasing neurons were previously identified [12, 13, 23], their cellular features and functional consequences remain to be fully clarified [13, 24, 25]. Their functional roles are just beginning to be elucidated by conditional deletion of *Slc17a6* in VTA dopaminergic neurons or by targeted analysis of *Slc17a6*/VGLUT2^+^ neurons in the VTA [26–28]. However, these studies were unable to target the co-releasing neurons selectively and failed to delineate their impact on synaptic plasticity and animal behaviors. We identified *Wwox* as a potential marker gene for these co-releasing neurons, which may enable them to be modulated in cell-type-specific and temporally dependent fashions both in vitro and in vivo.

Previously, Wwox was shown to act as a tumor suppressor whose loss of heterozygosity and chromosomal rearrangement have been detected in various cancers including ovarian, breast, hepatocellular, and prostate cancers [29]. Upon its phosphorylation at Tyr33 in the WW domain, activated WWOX acquires enhanced interactions with various transcription factors including p53, c-Jun, TNF, p73, AP2 gamma, and E2f1. Recently, a number of studies have reported that Wwox plays important roles also in the brain, and its dysregulation leads to neurodegeneration [30]. For example, *Wwox* is downregulated in the hippocampi of patients with Alzheimer’s disease [31], and knockdown of *Wwox* in neuroblastoma cells and mice resulted in aggregation of amyloid β and Tau [32]. However, the potential roles of *Wwox* in the VTA have been rarely investigated. WWOX binds and co-translates with many transcription factors to relocate to the nucleus to enhance or block neuronal survival under physiological or pathological conditions [33]. Our finding suggests that *Wwox* can be induced highly in dopamine- and glutamate-co-releasing neurons and selective targeting of these co-releasing neurons using *Wwox* may provide new insights into the roles of these neurons in neuronal survival in the VTA, as well as animal behaviors associated with the VTA.

The number of genes with the expression intensity available in the ABA continues to increase, which should lead to more comprehensive searches of marker genes. Moreover, the gene expression intensities from the sagittal section datasets can be combined with those from the coronal section datasets, and our analytical approach can be applied to the combined gene expression profiles. Genes that show specific expression in neuronal subpopulations consistently in both coronal and sagittal section datasets could be considered as more reliable candidates. Therefore, our analytical approach is widely applicable to the identification of various cellular marker genes in various cellular contexts and brain areas.

## Abbreviations

ABA: Allen Brain Atlas
CHRNA6: Cholinergic Receptor Nicotinic Alpha 6 Subunit
GAD67: Glutamate decarboxylase 67 which encoded by *Gad1*
IHC: Immunohistochemistry
ISH: in situ hybridization
MOC: Mander’s overlap coefficient
P2RY14: Purinergic Receptor P2Y14
SEM: Standard error of the mean
*Slc17a6*: Solute Carrier Family 17 Member 6
SNpc: Substantia nigra pars compacta
SNpr: Substantia nigra pars reticulata
TH: Tyrosine hydroxylase
VGLUT2: Vesicular glutamate transporter 2 which encoded by *Slc17a6*
VTA: Ventral tegmental area
WWOX: WW domain-containing oxidoreductase
